# Age patterns and transmission characteristics of hand, foot and mouth disease in China

**DOI:** 10.1186/s12879-016-2008-y

**Published:** 2016-11-21

**Authors:** Jijun Zhao, Fachun Jiang, Lianfa Zhong, Jianping Sun, Junhang Ding

**Affiliations:** 1 Complexity Science Institute, Qingdao University, Qingdao, Shandong China; 2 Qingdao Center for Disease Prevention and Control, Qingdao, Shandong China; 3 College of Automation and Electrical Engineering, Qingdao University, Qingdao, Shandong China

**Keywords:** Hand foot and mouth disease, Force of infection, Average age at infection, Reporting rate

## Abstract

**Background:**

Hand, foot and mouth disease (HFMD) has circulated in China and caused yearly outbreak. To understand the transmission of the disease and to assess the spatial variation in cases reported, we examined age-specific transmission characteristics and reporting rates of HFMD for 31 provinces in mainland China.

**Methods:**

We first analyzed incidence spatial patterns and age-specific incidence patterns using dataset from 2008 to 2012. Transmission characteristics were estimated based on catalytic model. Reporting rates were estimated using a simple mass action model from “Time Series Susceptible Infectious Recovered” (TSIR) modeling.

**Results:**

We found age-specific spatial incidence patterns: age-specific proportions of HFMD cases varied geographically in China; larger case percentage was among children of 3–5 years old in the northern part of China and was among children of 0–2 years old in the southern part of China. Our analysis results revealed that: 1) reporting rates and transmission characteristics including the average age at infection, the force of infection and the basic reproduction number varied geographically in China; 2) patterns of the age-specific force of infection for 30 provinces were similar to that of childhood infections in developed countries; the age group that had the highest infection risk was 3–5 years old in 30 provinces, and 10–14 years old in Tibet; 3) a large difference in HFMD transmission existed between northwest region and southeast region; 4) transmission characteristics determined incidence patterns: the higher the disease transmission in a province, the earlier the annual seasonality started and the more case percentage was among children 0–2 years old and less among 3–5 years old.

**Conclusion:**

Because HFMD has higher transmission than most childhood infections reported, high effective vaccine coverage is needed to substantially reduce HFMD incidence. Control measures before the vaccine implementation should focus on 2–6 years old children in 30 provinces and 10–14 years old children in Tibet.

**Electronic supplementary material:**

The online version of this article (doi:10.1186/s12879-016-2008-y) contains supplementary material, which is available to authorized users.

## Background

Hand, foot and mouth disease (HFMD) is a childhood infectious disease that mainly circulates in Asia-Pacific region, including Singapore, Taiwan, Hong Kong, Japan, Thailand and China [1–6]. In China, the disease has spread into all provinces of the country and there have been annual HFMD outbreaks nationwide since 2008. Causing millions of illness and hundreds of deaths every year [7] and without efficacious vaccines [8], HFMD has been a growing public health problem in China. Most research effort about HFMD has focused on effects of meteorological factors on the incidence seasonality [1, 9, 10], determinants of nationwide variation of report cases [1, 11], the spatio-temporal clustering in a locale [12], the development and efficacy of vaccines [13, 14], clinical features and diagnosis [15], and analyses of virology and pathogenesis [16]. In order to plan control measures to ease or even to eliminate HFMD, we need to understand factors that determine the incidence, the transfer and the persistence of the disease. Hence it is important to estimate transmission characteristics, such as the force of infection, the average age at infection and the basic reproduction number. For a childhood disease that most transmission happens among children, the age acts as the major determinant of a transmission risk. It is necessary to obtain profiles of age-specific transmission characteristics. Age related characteristics of major childhood diseases in developed countries have been well studied and documented by using pre-vaccination era data [17, 18]. Although age-stratified incidence proportions of HFMD was reported [1], there has been no age-specific transmission characteristics reported up to now.

The force of infection is the per capita rate at which susceptible individuals become infected [19]. The age-specific force of infection tells us risks of infection at specific ages. The basic reproduction number, *R*_0_, which quantifies transmission potential, is the average number of secondary cases resulting from an average primary case in a totally susceptible population [19]. High *R*_0_ can produce large epidemic outbreaks. Once we have profiles of the force of infection, transmission potentials among and between age groups can be obtained. Besides assessing the spread of the disease and evaluating impacts of probable interventions, these profiles can be further utilized in age-structured transmission models. Age-structured effects on the childhood disease transmission, such as the prevalence periodicity and the changing of epidemiology of disease, have been reported in a number of studies [20, 21]. As with many other childhood infectious diseases, age-structured effects likely play an important role in HFMD transmission dynamics.

Age-specific transmission characteristics of childhood diseases in China have rarely been studied due to the lack of consistent large scale data. To obtain transmission characteristics of childhood infections, pre-vaccination age-stratified case reports or serological data are important sources of information. Since May 2008, age-stratified report cases of HFMD are available from CDC China [7], making it possible to obtain transmission characteristics for HFMD. As will be shown in this paper, the consistent annual seasonality, the consistent case percentage among ages and the consistent age-specific force of HFMD infection from year to year mean that the HFMD transmission system is at its equilibrium in China. This offers an unique opportunity to improve our current understanding of the HFMD transmission and to sketch the first profile of age-specific transmission characteristics of childhood diseases in China.

Magnitudes of seasonality of HFMD varied greatly from south to north and from east to west in China showing spatial patterns [11, 22]. The spatial variation in HFMD occurrence has been associated with meteorological factors [1, 22–24], or social factors [11, 25]. Disagreements exist about the type of factors that is more responsible for HFMD occurrence in China. The geographical variation in the number of reported cases suggests two causes: one is the national variation in transmission characteristics, the other one is the national variation in reporting rates. Reporting rate is the percentage of infections that are reported to the surveillance system. Spatial heterogeneities of transmission characteristics were observed across European countries for childhood diseases due to large socio-economical differences [26–28]; large variations in reporting rates were also reported in Europe and Mexico [29, 30]. The spatial variation in magnitudes of HFMD cases reported in China may caused by the national variation in reporting rates and/or the variation in the transmission.

A large variation in reporting rates across the country would greatly affect the HFMD incidence or cases reported. Reporting rates have been rarely studied for HFMD in China, hence need to be estimated. Regarding the associations of meteorological or social factors with HFMD cases reported, we should aware of the limitations of these methods when reporting rates were not been considered. Geographically, China spans across several climatic zones and there are large socio-economical differences between provinces. Hence, transmission characteristics of HFMD might have national heterogeneity as a result of geographical differences in terms of the climate and socio-economic conditions. Spatial patterns of transmission characteristics need to be deeply examined such that determinants of HFMD transmission can be investigated and that targeted interventions can be made for different areas. Besides, national variation in transmission characteristics may help to explain spatial incidence patterns or age-specific incidence patterns.

In this paper, we aim to examine transmission characteristics of HFMD in China, including the force of infection, the average age at infection. By comparing patterns of transmission characteristics across the country, we identify degrees of variations of HFMD transmission in China. We do not explore in this paper which social or climatic factors cause the variation of HFMD transmission, but we use the transmission variation to explain age-specific incidence patterns and observed spatial occurrence patterns. We use our results to give advice on age groups with high infection risk in different areas, and we give advices on minimum coverage rates once a vaccination can be implemented. The estimated age-specific transmission characteristic parameters can be provided as primary parameter values for age-specific mathematical models that could be used to analyze causes of seasonality patterns and to evaluate efficacies of control measures.

## Methods

Mainland China has 31 provinces, municipalities and autonomous regions. Hong Kong, Macau and Taiwan were not included. The four municipalities include Beijing, Tianjin, Shanghai and Chongqing. Autonomous regions, including Tibet, Inner Mongolia, Guangxi, Ningxia and Xinjiang, have higher population of minority ethnic groups. To simplify our expression, we will not distinguish provinces, municipalities, and autonomous regions in this paper and they are all called “provinces”. For provinces and their abbreviations, please refer Table 1.

**Table 1:** Climate zone, abbreviation, reporting rate, average age at infection, basic reproduction number, vaccine coverage and target ages for 31 provinces in China from 2008–2011

	Province	Abbreviation	Report rate (%)	*A* ^a^	*R*_0_^b^	*p*_*c*_^c^ (%)	Target ages (years old)
Plateau zone	Tibet	TB	2.00	4.7	15.8	94	4, 10–14
	Qinghai	QH	1.79	3.8	19.5	95	3–6
Middle temperate zone	Heilongjiang	HLJ	6.50	3.9	19.3	95	3–6
	Jilin	JL	12.28	3.8	19.8	95	3–6
	Inner Mongolia	IM	9.8	4.2	18.0	94	4–6
	Liaoning	LN	11.96	3.8	19.7	95	3–6
	Ningxia	NX	7.61	3.5	21.5	95	3–6
	Gansu	GS	3.46	3.9	19.3	95	4–5
Mixer zone	Xinjiang	XJ	1.99	3.8	19.5	95	3–5
Warm temperate zone	Beijing	BJ	21.97	3.6	20.7	95	3–5
	Tianjin	TJ	18.97	3.8	19.9	95	4–5
	Hebei	HeB	9.35	3.0	25.2	96	2–6
	Shanxi	SX	7.63	3.4	22.2	96	3–5
	Shangdong	SD	10.48	3.1	24.4	96	2–6
	Henan	HeN	7.34	2.6	29.2	97	2–6
	Shaanxi	ShX	11.17	3.0	24.6	96	2–5
Subtropical zone	Jiangsu	JS	12.65	3.2	23.3	96	3–5
	Anhui	AH	10.45	2.8	26.7	96	2–6
	Shanghai	SH	21.09	3.4	21.9	95	3–6
	Hubei	HB	9.85	2.9	26.2	96	2–5
	Sichuan	SC	4.88	3.0	25.2	96	2–5
	Zhejiang	ZJ	18.13	3.0	25.2	96	2–6
	Chongqing	CQ	6.07	3.1	24.0	96	3–5
	Jiangxi	JX	5.27	2.5	30.1	97	2–6
	Hunan	HuN	11.18	2.7	27.9	96	2–5
	Guizhou	GZ	5.36	3.0	25.0	96	2–5
	Fujian	FJ	11.79	2.7	27.2	96	2–6
	Yunnan	YN	6.37	3.2	23.5	96	3–6
	Guangdong	GD	18.24	2.9	25.9	96	2–5
	Guangxi	GX	19.96	2.8	27.0	96	2–6
Tropical zone	Hainan	HN	24.83	2.6	28.7	97%	2–5

^a^The average age at infection. ^b^The basic reproduction number assuming mixing is homogeneous. ^c^The critical immunization coverage rate

We utilized three data sets for our study. Data set I was provided to us by The Data-center of China Public Health Science. It consisted of weekly case reports of HFMD for 31 provinces in mainland China from January 2008 to December 2011. Case reports of each province in data set I were stratified into 24 age groups. The first 10 age groups were for ages from 0 years old to 9 years old; the other 14 age groups were for ages of 10–14 years old to 75–79 years old that had five years included in each group. China started national enhanced surveillance system since the first week of May 2008 [1, 7], hence we used data in data set I started from May 2nd 2008. We downloaded data sets II and III from the website of The Data-center of China Public Health Science [7]. Dataset II consisted of age-stratified monthly case reports of the entire country (however not divided for provinces) of year 2012. The 24 age groups are the same as that in data set I. Dataset III consisted of monthly case reports of year 2012 for 31 provinces however were not stratified into age groups. We mainly used data set I for our analysis, however data sets II and III were utilized where they were useful. Data sets I and III were used for the exhibit of time series of incidence for 31 provinces from 2008 to 2012, and for estimates of reporting rates for 31 provinces. Data set I was used for analysis of age-specific incidence patterns, age-specific force of infection and the average age at infection for 31 provinces from 2008 to 2011. Data sets I and II were used for estimates of age-specific force of infection for the country for year 2008 to 2012.

Birth rates and populations from 2008 to 2011 for provinces that were utilized for calculating reporting rates were obtained from National Bureau of Statistics of China [31].

We first present incidence patterns and age-specific incidence patterns nationwide by analyzing the age-stratified data. These patterns are later explained by variation in transmission characteristics.

We used a simple mass action model [32], which was from “Time Series Susceptible Infectious Recovered” (TSIR) modelling, to estimate the average reporting rate from year 2008 to 2012 for each province. Here we only give a brief equation of the method. Interested readers please refer Jackson et al. [32]. To show that there were large differences in reporting rates among provinces, reporting rates were assessed for all 31 provinces.

The reporting rate *ρ* can be estimated from the gradient 1/*ρ* of the fitted line of the following equation:

1 $$ {\displaystyle \sum_{k=1}^{t-1}B\left(k-d\right)}=\frac{1}{\rho }{\displaystyle \sum_{k=1}^t{Y}^r(k)+D(t)}-D(0), $$

where, *Y* ^*r*^(*k*) is the number of infected that are reported at time *k*; ∑_*k* = 1_^*t*^ *Y* ^*r*^(*k*) is the cumulative number of infected reported by the given time; *B*(*t*) is the number of births at time *t*; ∑_*k* = 1_^*t* − 1^ *B*(*k* − *d*) is the cumulative number of births by the given time; *d* is the duration of maternally derived immunity assumed to be 6 months, *D*(*t*) is the residuals from the fitted line.

We estimated HFMD transmission characteristics from age-stratified incidence data. We used a standard approach, which modelled infection process as a catalytic model. The literature on this standard approach is well established and the approach has long been applied to infectious diseases including major childhood transmission diseases [18–20, 30, 33].

The age-specific force of infection can be estimated from serological data or notification data. Suitable serological data of HFMD in China were not available, hence we used notification data to estimate. We first estimated the force of infection of 24 age groups for the country level using data sets 1 and 2 for each year from 2008 to 2012. Given notification data, we can calculate the force of infection in each age group according to Anderson and May [34]: *λ*(*i*) = −ln[(1-*p*_*i*_)/(1-*p*_*i*-1_)]/(*a*_*i*_-*a*_*i*-1_), where *λ*(*i*) is the average force of infection of age group *i*; *a*_*i*_ is the ending age of the age group *i*, hence *a*_*i*_-*a*_*i*-1_ is the width of the age group *i*, *p*_*i*_ is the proportion of cases by age group *i*; *n* is the number of age groups, here *n* = 24. To account for maternally derived antibodies, *a*_0_ was set as 0.5. Here we have the assumption that reporting rates are constant with respect to age and that the population is constant with age. These assumptions assume that the data reflect the endemic equilibrium.

We then assessed the age-specified force of infection for each province to compare the distribution patterns of the age-specific force of infection.

To calculate the average age at infection, *A*, we used the method in Anderson and May [34]:

2 $$ A=\left[{\displaystyle {\int}_0^La\lambda (a)X(a)da}\right]/\left[{\displaystyle {\int}_0^L\lambda (a)X(a)da}\right], $$

where *L* is the life expectancy, and *X*(*a*) is the number of Susceptible at age *a* and can be calculated based on catalytic model. The average age at infection was calculated at national level and for 31 provinces.

Finally, we examined the association of the average age at infection with identified incidence patterns to show that spatial patterns and age-specific incidence patterns are determined by the variation of the transmission of the disease.

## Results

### Incidence patterns and age-specific incidence patterns

**Fig. 1:**
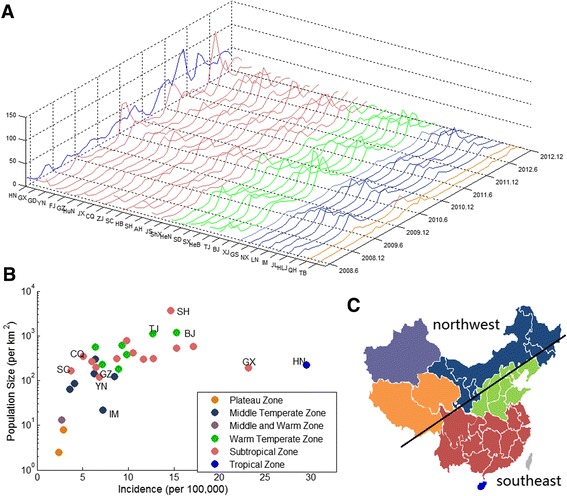
Incidence of HFMD for 31 provinces in China from May 2008 to December 2012. **a** Incidence of 31 provinces. Provinces from left to right were ranked by climate zone that the province is in and the latitude of province captical if provinces were in the same climate zone. **b** Scatter plot of average incidence and population density. Since many studies associate incidence with climate and population density, it is reasonable to include climate zone information and population density into the exhibit of incidence. **c** Provinces colored according to which climate zones they belong to. The country were divided into northwest and southeast regions

Figure 1a shows time series of incidence for provinces in China from May 2008 to December 2012. Figure 1b shows the scatter plot of incidence versus population density for provinces in different climatic zones. China can be roughly divided into two parts according to its humidity and temperature: the drier and colder part in the northwest and the more humid and warmer part in the southeast (Fig. 1c). The southeast part includes tropical, subtropical and warm temperate zones; the northwest part includes middle temperate zone, the plateau zone and the middle and warm mixture zone. See Table 1 for climatic zones for provinces.

Nationally, there were annual peaks of incidence during spring or early summer. Some provinces especially those in the southern China had a minor peak during October. The spring peak of incidence started earlier in south China (April or May in Hainan, the only province in tropical zone), and started relatively late in north (July in Heilongjiang, the most north province in middle temperate zone). Tibet’s seasonality pattern was different from the seasonality pattern of all other provinces: incidence peaked only in October rather than in spring or early summer. The above seasonal patterns were similar to that reported in Xing et al. [1] which was based on surveillance and clinical cases in 31 provinces. Since we examined the incidence instead of cases to consider population size effects, relative amplitude of incidence for provinces maybe different than that in Xing et al. [1].

**Fig. 2:**
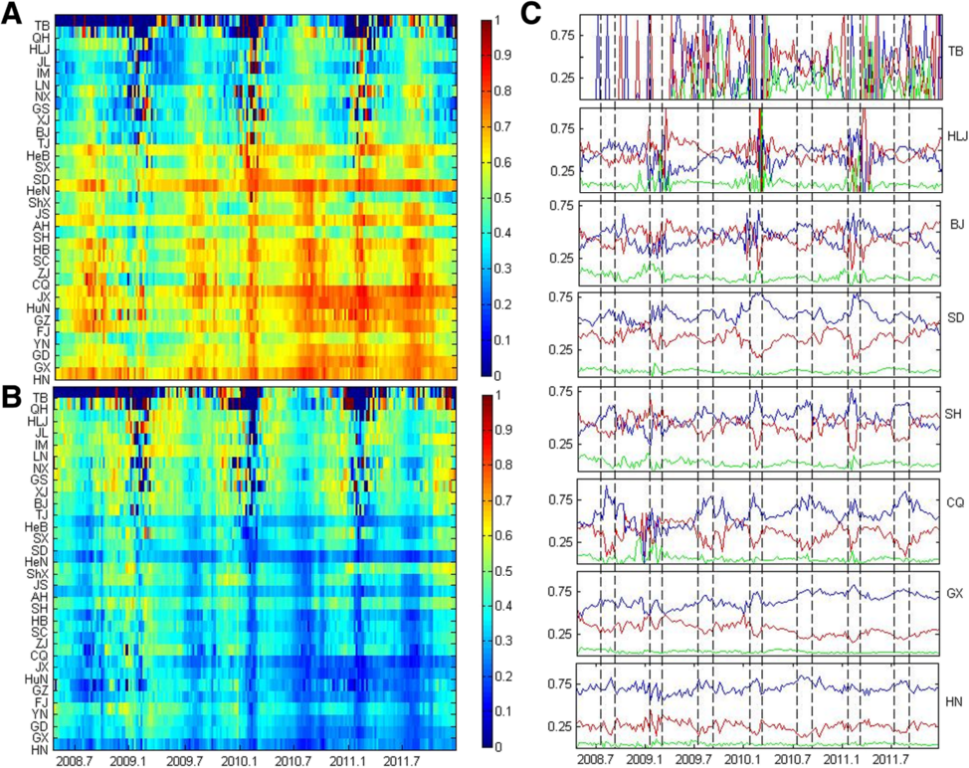
Heatmaps of age-specific incidence of HFMD for 31 provinced in China from January 2008 to December 2011. **a** Heatmap of case percentage among 0–2 years old for 31 provinces. **b** Heatmap of case percentage among 3–5 years old for 31 provinces. **c** Case percentage among age groups of 0–2 years old (blue line), 3–5 years old (red line) and 6–19 years old (green line) of some selected provinces. Winter and summer vacations are shown in narrow bands. Winter and summer vacation times in China are: 1 month during January or February depending on Chinese lunar new year, and from early July to the end of August

Age-specific proportions of HFMD cases varied geographically in China (Fig. 2). Case percentage among age groups of 0–2 years old (Fig. 2a) and case percentage among age groups of 3–5 years old (Fig. 2b) shows the seasonality of case percentage of young ages. Figure 2c shows case percentage of age groups for some selected provinces. Cases were up 95% among children younger than 6 years old in south provinces and 85% in northwest provinces. The case percentage among children of 0–2 years decreases from around 70% in Hainan to around 40% in Heilongjiang, and in turn, the case percentage among children of 3–5 years increases from 25% to around 45%. The age-specific incidence patterns were consistent from 2008 to 2011. Children of 3–5 years old are in kindergarten age.

### Reporting rates

The great distinction in reporting rates for provinces from 2008 to 2012 were found: from around 2% to around 25% (Table 1). Beijing, Tianjin and Shanghai that had highest population densities and were economically developed had reporting rates around 20%. Besides, provinces in the southernmost part of China, such as Hainan, Guangdong and Guangxi, had reporting rates from 18 to 25%. Provinces in plateau zone and mixture zone had the lowest reporting rates.

### The force of infection

**Fig. 3:**
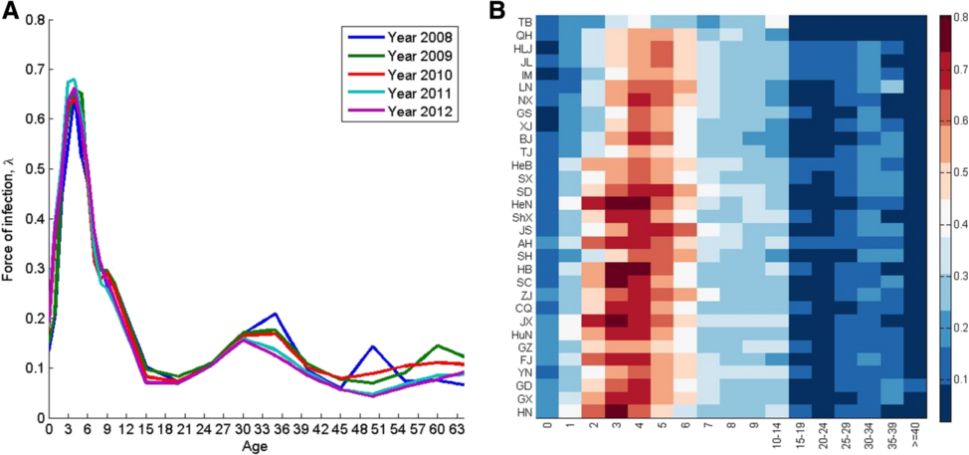
Age-specific force of HFMD infections at the country level and in 31 provinces in China. **a** Age-specific force of infection for HFMD in China from 2008 to 2012. **b** Heatmap of averaged age-specific force of infection for HFMD in 31 provinces from May 2008 to December 2011

Patterns of the age-specific force of infection for HFMD in China were consistent from year to year (Fig. 3a). The force of infection increased rapidly with age when children were under 3 or 4 years old, and declined into a plateau during adolescence and then had a subsequent small peak when people got into 25 years old until 39. The subsequent small peak was probably because that parents of 25 to 39 years old were infected by their children, which was similar to the results in a study of pertussis [20]. The force of infection of 3–4 years old was around 65% per year. High level of force of infection means high risk of being infected.

An extensive investigation of forces of infection was made at the spatial level. A heatmap of averaged age-specific forces of infection of year 2008 to 2011 for provinces was constructed (Fig. 3b). Patterns of age-specific force of infection for most provinces were similar to the pattern in the country level, except Tibet. There were variations among peak ages and among peak levels of age-specific force of infection for provinces. Provinces in the southeast part of China had a relatively higher level of peaks than those in the northwest part. Peaks were relatively at younger age (3 years old) in provinces in south, and were at older age (5 years old) in north and west provinces. Surprisingly, the spatial patterns of the force of infection with respect to ages (Fig. 3b) looked similar to the spatial pattern of incidence seasonality with respect to months of the year in Fig. 4b in Xing et al. [1]. Provinces whose seasonality started early in spring had the age-specific force of infection peaking at younger ages. We will associate the seasonality peak time with a summary statistics, the average age at infection of the disease, in the next subsection.

**Fig. 4:**
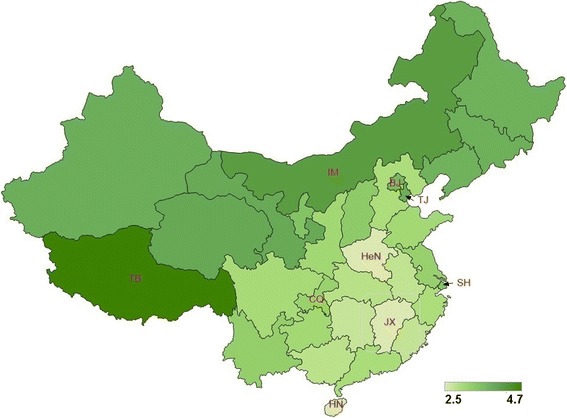
The average age at infection of HFMD in 31 provinces in China from 2008–2011. Figure was generated using Tableau Desktop version 8.3 (http://www.tableau.com/support/releases/8.3)

In Tibet, two age groups, 4 years old and 10–14 years old, had high force of infection; children ages 10–14 years had the highest force of infection.

Jiangxi and Henan were two provinces that had the highest peak amplitude of the force of infection, and their peak age (2–4 years) was younger than their surrounding provinces. This suggests that in Jiangxi and Henan, children ages 2–4 had higher risk than other ages, while in their surrounding provinces children ages 3–5 years had higher risk than other ages. The force of infection distribution in age can be used to target age groups for control measures. In Tibet, 10–14 years had highest risk, hence together with 4 years old, they are the target group. In other provinces in northwest China and the four large cities, 3–6 years old, that is the kindergarten children and the first year in primary school should be the target; all other provinces in the southeast part of China, the target should be 2–5 years, mostly are children in kindergarten age (see Table 1).

### The average age at infection, and the basic reproduction number

The average age of HFMD infection for the entire country from May 2008 to December of 2011 was 2.99. The average age at infection for 31 provinces show spatial patterns (Fig. 4 and Table 1). Large difference in average age at infection existed between the northwest region and the southeast region. Provinces in the southeast part of China had 0.92 (*p* = 5.9e-6) year lower average age at infection than provinces in the northwest part of China.

Average age at infection is a rough summary of the force of infection, hence provinces whose forces of infection peaked at younger age had lower average age at infection, such as Jiangxi and Henan. Lower value of the average age at infection suggests higher transmission rate of the disease. If we assume that individuals mix randomly, the basic reproduction number *R*_0_ can be calculated as *R*_0_ ≈ *L*/*A*, where *L* is the life expectancy of the country and *A* is the average age at infection [33]. Life expectancy in China reported in 2010 by National Bureau of Statistics of China was 74.8 years. *R*_0_ in the country level was 25, which was higher than *R*_0_s of most childhood transmission diseases calculated under the same assumption. *R*_0_s of provinces is listed in Table 1 ranging from 15.8 to 30. HFMD was more transmissible in the southeast part of China.

### Determinants of incidence patterns and age-specific patterns

In this subsection, we discuss that observed incidence patterns and age-specific patterns are determined by the variation in transmission characteristics. In this paper, we only demonstrated degrees of variation of HFMD transmission, and we did not investigate which factor (climate or socio-economical factors or their combinations) caused the variation of the HFMD transmission, and which factor caused the significant transmission pattern in Tibet. We will discuss determinant factors in our subsequent paper.

**Fig. 5:**
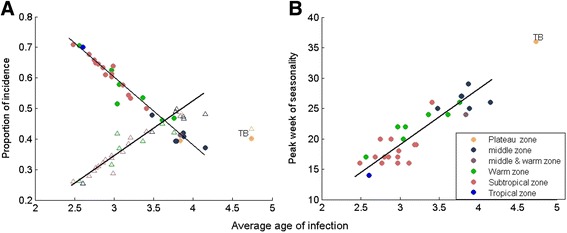
The relationship of the average age at infection with case percentage among age groups and timing of seasonality for 31 provinces in Chia from 2008–2011. **a** Average age at infection vs proportion of incidence. Color of symbols according to climate zone, with filled circles representing case percentage among children of 0–2 years old and open triangle representing case percentage among children of 3–4 years old. Regression lines exclude data of Tibet. **b** Average age at infection vs starting time of spring seasonality of incidence. Tibet have fall seasonality instead of spring seasonality of incidence, hence it was excluded from the regression line

The relationship of the average age at infection and the case percentage among age groups is shown in Fig. 5a. The case percentage among children of 0–2 years old decreased with the average age at infection, whereas the case percentage among children of 3–5 years old increased with the average age at infection. In the southern part of China, more proportion of infected were at age of 0–2 years old, while in the northern part of China, except Ningxia, more proportion of infected were at age of 3–5 years old. Since transmission characteristics of Ningxia was similar to provinces in the warm temperate zone, Ningxia behaved more like those in the warm temperate zone.

As seen in the previous subsection, the transmissibility of the disease should affect the peaking time of HFMD seasonality. Figure 5b shows the association between peak weeks of seasonality and the average age at infection. The more transmissible the HFMD (lower average age at infection) was in a province, the earlier the disease seasonality started there. The shift of the seasonality peaking time can also be explained by the age-specific force of infection: the younger the individual had the highest risk, the earlier the seasonality started.

The seasonality pattern of HFMD in Tibet was different than other provinces: incidence peak in October, 1 month after schools resume. Meanwhile, the age distribution of the force of HFMD infection in Tibet is quite different than the other 30 provinces in China. In Tibet, the 10–14 year age group has the highest force of infection hence the highest risk. Many factors, such as population density, economic development or social behavior had been shown to be associated with nationwide variation in HFMD occurrence [11, 23, 25]. These factors may cause the significant difference in the distribution of the force of infection in Tibet, which should be deeply investigated. Here we only show the possible connection of the force of infection pattern with the observed seasonality pattern of HFMD in Tibet. The seasonality peaked on October in Tibet may be the result of highest risk of infection of 10–14 years old school children.

## Discussion

This paper estimated some key parameters of HFMD transmission that can be applied as initial parameter values to dynamic models where uncertainties of these parameters can be assessed and potential causes of transmission patterns can be found. We systematically studied geographical patterns of estimates of these epidemiology parameters across China. The reporting rate and transmission characteristics such as the age-specific force of infection, the average age at infection and the basic reproduction number of HFMD varied across the country. The variability in HFMD transmission in different area has important implications for the design of control measures.

The reporting rate of HFMD in China varied from 2 to 25%. Great spatial heterogeneities in reporting rates of childhood infections in pre-vaccination era were also reported in Europe (from 2 to 12%) [29] and Mexico (from 0.3 to 18%) [30]. The reporting rate of HFMD in China has been rarely studied. In one study, the reporting rate of Jiangsu was 11.2% [35]. The reporting rate of Jiangsu in our results (12.6%) was in agreement with it.

Jiangxi and Henan had similar average age at infection as Hainan and Guangxi, however their incidence reported were much lower. A possible reason was that Jiangxi and Henan had much lower reporting rates (6–7%). Without considering large variation in reporting rates, analysis results of determinant factors by comparing reported incidence maybe significantly distorted. The force of infection and the average age at infection are not affected by the variation in reporting rates among provinces, hence can be used to find driving factors of the HFMD transmission.

Patterns of age-specific force of infection of the 30 provinces (except Tibet) in China were similar, and were in agreement with patterns of other childhood transmission diseases in developed countries. However, the magnitude of the force of HFMD infection estimates in China were much higher, and the peak age was much younger. Peak values of the force of infection of measles, mumps, and rubella in Europe were less than 50, 30 and 25% per year respectively [26]; that of pertussis in Europe was less than 45% per year [20]. The estimate of the force of HFMD infection was as high as 68% per year in the country level, and could be as high as 80% in some provinces. In Europe, the force of infection of childhood diseases in pre-vaccination era peaked at older ages: measles and rubella 5–10 years old, mumps 5–7 years old [18, 26], pertussis around 6 [20]. These patterns were consistent in varies locations and time. Regarding HFMD, the higher risk was in younger age children, especially children in kindergarten. In China, children stay in kindergarten full day. Classrooms in kindergarten may be not crowded as in school, but longer time in kindergarten increases contact rate among kindergarten children. Due the fact that kindergarten aged children have higher HFMD infection risk and may have high contact rates, HFMD control measure should focus on them.

It has been theoretically predicted and documented that shifts in the average age at infection and in the age-specific case proportion could be caused by vaccinations of diseases [20, 33, 36]. Our study is the first systematic comparison of the average age at infection and the age-specific incidence in a large geographical environment before a vaccination is implemented. This study shows that variation in disease transmission can explain spatial incidence patterns and spatial age-specific incidence patterns. The higher the HFMD transmission, the more case proportion is among 0–2 years children, and the less among 3–5 years children; the higher the HFMD transmission, the earlier the annual spring seasonality starts and peaks each year.

The basic reproduction number *R*_0_ estimated in this paper is crude, however we can see where HFMD transmission stands at by putting *R*_0_ of HFMD along with *R*_0_s of other childhood infections that were estimated based on a similar assumption. Besides, relative magnitude of *R*_0_ for provinces can be compared to see the spatial transmission patterns across the country. Our estimates of *R*_0_ (15.8–30 for different provinces) in mainland China during 2008–2011 were much larger than the estimates of *R*_0_ of HFMD in Taiwan (1.37, 95% CI: 0.24–5.84) for year 2008 [37]. Initial proportion of susceptible individuals, *S*_0_, in the model of Lai et al. [37] was estimated as 45% based on seropositive rates in 1999 after an outbreak in Taiwan. Compared with seropositive rates of Luan city in central China in 2010 after the 2008 outbreak [38], seropositive rates for similar ages in Taiwan were much lower. Lower seropositive rates in Taiwan means that the transmission of HFMD in Taiwan in 1999 is lower than the transmission in mainland China in 2010. The initial value of *S*_0_ can highly affect the estimates of *R*_0_ [19]. Our estimates of initial *S*_0_ could be as smaller as 1/10 of that in Taiwan (unpublished observations). It is reasonable that our estimates of *R*_0_ is much higher than that in Taiwan. The basic reproduction numbers were estimated to be 5.48 and 2.5 for EV71 and CVA16 respectively among children in kindergarten in Hong Kong. The estimates is for transmission rate among children in kindergarten, not for the entire population. Estimates of *R*_0_ of population should be larger than the basic reproduction number among kindergarten children. *R*_0_ of childhood infections are usually high. Estimated basic reproductive numbers for measles, whooping cough, chickenpox, mumps and rubella were 16–18, 16–18, 10–12, 11–14, and 6–7 [19, 33] respectively for developed countries. *R*_0_ of a same disease in a developing country was even higher. The higher the value of *R*_0_, the lower the average age at infection. The average age at infection of measles in developed countries were 4–6 years old, and that of HFMD was around 3. It is reasonable that *R*_0_ of HFMD is larger than 16–18. It is highly unlikely that *R*_0_ of HFMD is just a little bit higher than 1 in mainland China.

Before efficacious vaccination is implemented, control measures should focus on targeted age groups for each provinces as discussed in the Results section. Control measures, such as improving hygienic environment in kindergarten, kindergarten closing and quarantine, can reduce HFMD transmission when there is epidemic, however an effective vaccination should be a better control measure to eliminate the disease because vaccination can largely reduce the force of infection. The HFMD Vaccine is under clinical trial [13, 14]. Hopefully, vaccination maybe put into use in the very near future. The critical immunization coverage of HFMD, denoted *p*_*c*_, can be derived by equation [19]: *p*_*c*_ = 1-1/*R*_0_, that should be above 96% nationally. For provinces such as Hainan, should be >97%, Tibet above 94%. With higher *R*_0_, higher vaccination coverage needed to achieve control and elimination. Although the estimates of *R*_0_ in this paper were not based on age-specific *R*_0_, the crude estimates still can give us guidelines on immunization coverage rate before we know the exact contact matrix of HFMD transmission in China. Because HFMD has lower average age at infection and a higher basic reproduction number than most childhood infections reported [19, 33], transmissibility of HFMD is among the highest. This means it will take longer time for HFMD than other childhood infections to be substantially reduced to a low level and will be very hard to be eliminated. The critical immunization coverage is the product of the actual vaccination coverage and the vaccine efficacy. The vaccine efficacy against EV71-associated hand, foot, and mouth disease was 94.8% as reported in a phase 3 trial [13]. If all newborns were immunized with a single dose, the critical immunization coverage still can not be achieved for most provinces.

The age-specific incidence has seasonality. This reflects that the transmission rate varies with the opening and closure of kindergartens. The seasonality of transmission rate does not change the average value of transmission rate [19], but it may have effect on the seasonality of HFMD incidence. The spring seasonality of report cases was thought due to climatic factors such as the temperature and humidity [1]. Reasons of the autumn peak are not clear [1]. As a childhood transmission disease, contact among children have significant role in the transmission. Seasonality of some virus infectious diseases in childhood have been shown due to opening and closure of schools. The kindergarten opening and closure may have effects on the spring and autumn peaks of HFMD in China. A seasonality math model is needed to further investigate the effects of kindergarten aggregation.

In the estimation of the age-specific force of infection, we assumed that reporting rates were the same for all age groups. This may not be the situation in reality where adults may have a lower reporting rate. This may cause a bias in the estimation that the force of infection of adults might be higher than estimated. The force of infection (except in Tibet) increased rapidly under 3–5 years old and then decreased after that. The changing mainly happened before age 10. It is unlikely that the reporting rate differ greatly in this age range, hence the bias will not affect the overall pattern. Besides the effect of reporting rates of age groups, the ratio of asymptomatic to symptomatic infection by age group have important implications in understanding HFMD transmission. All these call for complete study of serological data.

Simulated results showed that the standard approach gave mean estimates of the age-specific force of infection that appear unbiased for the true force of infection [39]. There are situations when estimates from the standard approach have bias, for example when a disease has extreme irregularities of outbreaks (frequent local extinction and outbreaks that can vary over many orders of magnitude) [39]. In this kind of situation, the standard approach should be corrected [39]. HFMD is not in this kind of situation. Estimates of transmission characteristics from standard approach have important applications: the age-specific force of infection can be used to identify age groups that have higher infection rates [19, 33] and can be applied as initial parameter values to dynamic models [21]; the average age at infection can be analyzed for impact factors of disease transmissions [40].

This is only a primary analysis of the transmission characteristics of HFMD in China. We calculated *R*_0_ by method that was direct, simple and could utilize transmission characteristic values that were estimated in our study. Age-specific *R*_0_s will be intensively investigated based on possible contact matrices in our future study. We did not discuss determinant factors that cause the nationwide variation in the transmission characteristics. This is our objective of our next manuscript. We put our efforts into parameter estimation from notification data assuming each provinces are autonomous. Transportation and seasonal immigration among provinces can be analyzed using spatially structured metapopulation models in the future.

## Conclusion

In China, form 2008 to 2011, the reporting rate and transmission characteristics of HFMD vary geographically. Children of 3 to 5 years old had the highest force of HFMD infection in 30 provinces. Variation in transmission characteristics determined spatial incidence patterns.

## Additional files

Additional file 1: Age proportion. The HFMD case percentage of 26 age groups for 31 provinces in China. Description of data: This data file is about the HFMD case percentage of 26 age groups for 31 provinces in China. Age groups of 1 to 10 are for ages from 0 years old to 9 years old; age groups 11 to 25 are for ages of 10–14 years old to 80–84 years old that have 5 years included in each group; age group 26 is for ages that are older than 85. Age groups 1 to 24 were used in our analysis. (XLS 40 kb)
Additional file 2: Force of Infection of provinces. The age-specific force of HFMD infection for 31 provinces in China. Description of data: This data file records the age-specific force of HFMD infection that were estimated in our study. (XLS 32 kb)
Additional file 3: Incidence Reported. Monthly reported HFMD cases for 31 provinces in China. Description of data: The monthly reported HFMD cases for 31 provinces in China can also be downloaded from the Data-center of China Public Health Science. (XLS 54 kb)

## Abbreviations

HFMD: Hand, foot and mouth disease
TSIR: Time Series Susceptible Infectious Recovered
